# Clonal hematopoiesis of indeterminate potential (CHIP) and cardiovascular diseases—an updated systematic review

**DOI:** 10.1186/s43141-021-00205-3

**Published:** 2021-07-19

**Authors:** Nagendra Boopathy Senguttuvan, Vinodhini Subramanian, Vettriselvi Venkatesan, T. R. Muralidharan, Kavitha Sankaranarayanan

**Affiliations:** 1 Department of Cardiology, Sri Ramachandra Institute of Higher Education and Research, Chennai, Tamil Nadu 600116 India; 2 Department of Human Genetics, Sri Ramachandra Institute of Higher Education and Research, Chennai, Tamil Nadu 600116 India; 3 Department of Cardiology, Sri Ramachandra Institute of Higher Education and Research, Chennai, Tamil Nadu 600116 India; 4 Ion Channel Biology Laboratory, AU-KBC Research Centre, MIT Campus of Anna University, Chennai, Tamil Nadu 600044 India

**Keywords:** Cardiovascular diseases, Myocardial infarction, Clonal hematopoiesis of indeterminate potential (CHIP), Mutations

## Abstract

**Background:**

Cardiovascular diseases (CVDs) are the leading cause of mortality in India. Residual risk exists in patients receiving optimal guideline-directed medical therapy. Possession of certain somatic mutations, at a variant allele frequency of ≥ 2% in peripheral blood, driving clonal expansion in the absence of cytopenias and dysplastic hematopoiesis is defined as clonal hematopoiesis of indeterminate potential (CHIP). Recently, it was found that carriers of CHIP had a higher risk to have coronary artery disease (CAD) and early-onset myocardial infarction. Association of CHIP with heart failure and valvular heart diseases is increasingly being considered. The common link that connects CHIP mutations and CVDs is inflammation leading to increased expression of cytokines and chemokines. We intended to do a systematic review about the association of CHIP mutations and CVD along with identifying specific CHIP mutations involved in increasing the risk of having CVDs.

**The main body of the abstract:**

We performed an extensive literature search in PubMed and Google Scholar databases. Out of 302 articles, we narrowed it down to 10 studies based on our pre-specified criteria. The methodology adopted for the identification of CHIP mutations in the selected studies included – whole-exome sequencing (*n* = 3), whole-genome analysis (*n* = 1), transcriptome profiling analysis (*n* = 1), whole-genome analysis (*n* = 1), and single-cell RNA-sequencing (*n* = 1). We found that the available literature suggested an association between CHIP and CVD. The most commonly described CHIP mutations in patients with CVD are *DNMT3A*, *TET2*, *ASXL1*, *TP53*, *JAK2*, and *SF3B.* We further analyzed the commonly mutated CHIP genes using bioinformatics tools. Protein function and interaction analysis were performed using the g: Profiler and GeneMANIA online tools. The results revealed significant bio grid interactions for molecular functions, biological processes, and biological pathways. Interaction analysis showed significant physical and co-expression interactions.

**Short conclusion:**

We conclude that there exists a significant association between CHIP mutations and CVD with *DNMT3A*, *TET2*, *ASXL1*, *TP53*, *JAK2*, and *SF3B* as the commonly implicated genes*.* The recognition of the link between CHIP and cardiovascular events will expand our understanding of residual risk and will open up new avenues of investigation and therapeutic modalities in the management of patients with CVD.

**Supplementary Information:**

The online version contains supplementary material available at 10.1186/s43141-021-00205-3.

## Background

CHIP is characterized by the absence of definitive morphological neoplasm and the presence of a somatic mutation associated with hematological neoplasia at a variant allele frequency of at least 2%. The hallmarks of CHIP are an absence of phenotypic variation in blood cells that implies hematological evidence; the presence of a somatic mutation that is associated with the hematologic condition, at a VAF of > 2%; and the lack of diagnostic symptoms that are associated with hematological cancer [1, 2].

## Main text

Atherosclerotic cardiovascular diseases (ASCVD) remain the major cause for mortality and morbidity globally [3]. World Health Organization reduces the burden of the same by 25% by 2025 [3]. Epidemiologic transition from communicable diseases to non-communicable diseases (NCDs) happens in India [4]. A significant amount of evidence exists to show an increased risk of CVD in south Asian immigrants and native south Asians as compared to Caucasians [5–7]. Proposed reasons being higher levels of small dense LDL [8], lower levels of HDL especially functional HDL, higher levels of lipoprotein(a), and higher prevalence of diabetes mellitus [7]. For the same BMI, south Asians have been found to have higher abdominal visceral fat leading to greater levels of insulin resistance [8–11]. Though lots of risk scores to predict CVD are available, it is Q-risk score [12] that give increased risk to south Asians. In 2018, ACC/AHA produced its updated blood cholesterol guidelines [13] that also scored increased risk to south Asians for having CVD. As compared to western countries, CVD in India involve younger patients, many patients had STEMI. They were less likely to receive evidence-based therapy and hence had higher mortality [14]. This shows there might be more than just conventional risk factors that may predispose south Asians for CVD. Among the non-conventional risk factors, lipoprotein(a) [15], homocysteine, plasminogen activator inhibitor-1, and fibrinogen have generated considerable interest [7]. Currently, the best possible way to decrease ASCVD is to reduce LDL levels [13], triglycerides using icosapent ethyl [16, 17], and controlling other traditional risk factors associated with ASCVD [13, 14]. A recently concluded trial showed a major adverse cardiac event (MACE) in 10% of patients who had been targeted to have a LDL between 25 and 50 mg using pro-protein convertase subtilisin kexin-9 (PCSK9) inhibitors during the follow-up 2.2 years. This highlights the importance of identifying residual risk for future MACE non-traditional and non-conventional risk factors for ASCVD.

Occurrence of somatic mutations in pre-leukemic driver genes that drive clonal expansion in the absence of malignancy can be considered CHIP. CHIP are often benign and do not progress; it could be considered as a precursor for hematological malignancies similar to monoclonal gammopathy of undetermined significance and monoclonal B cell lymphocytosis. Identification of such somatic mutations associated with cardiovascular diseases (CVD) may lead to a paradigm shift in the management of patients with CVD. We intended to do a systematic review about the association of CHIP mutations and CVD along with identifying specific CHIP mutations involved in increasing the risk of having CVDs.

## Methods

### Focused question

The research question were as follows

1. “Is there an association between CHIP and risk of developing CVD?”
2. “What are the CHIP mutations associated with an increase in the risk of developing CVD?”

### Eligibility criteria

Studies (case-control, cohort, cross-sectional design) that analyzed the association of CHIP mutations with CVD, genetic loci, and whole-genome/exome sequencing with CVD and CHIP with functional analysis were included. Those studies that had been conducted exclusively on animals, case reports, case series, non-research articles, and those not in the English language were excluded.

### Search strategy

An extensive literature search was performed on PubMed and Google Scholar databases using a combination of keywords “Clonal Hematopoiesis Of Indeterminate Potential; Cardiovascular Diseases; Coronary Artery Disease; Atherosclerosis; Stroke; Heart Failure; Myocardial Infarction; Aortic Stenosis; Premature Aging”. These keywords were combined to retrieve the abstracts (Table 1)

**Table 1:** Search strategy performed

Database	Search query	
PubMed	#1	(CLONAL HEMATOPOIESIS OF INDETERMINATE POTENTIAL)
	#2	((((((((CARDIOVASCULAR DISEASES) OR (CORONARY ARTERY DISEASE)) OR (ATHEROSCLEROSIS)) OR (STROKE)) OR (HEART FAILURE)) OR (MYOCARDIAL INFARCTION)) OR (AORTIC STENOSIS)) OR (PREMATURE AGING))
	#3	#1 AND #2
Google Scholar	Clonal Hematopoiesis Of Indeterminate Potential; Cardiovascular Diseases; OR Coronary Artery Disease; OR Atherosclerosis; OR Stroke; OR Heart Failure; OR Myocardial Infarction; OR Aortic Stenosis; OR Premature Aging	

#### Study selection

Two authors (Dr. NBS and VS) independently assessed the studies identified by the search strategy based on title, abstract, and keywords. The full text of the shortlisted studies meeting the inclusion criteria and those studies where the abstracts were ambiguous were obtained. Any disagreement between the two authors was settled by the third author (Dr.VV). Data extraction was performed by two authors (Dr. NBS and Dr. VV) independently. Data regarding the author’s name, study design, methodology adopted, sample size, mutations identified, and gene/genetic locus were obtained from the articles included for review. The following information was extracted from each study—author, year of publication, sample size, study design, and outcome. The references of the existing reviews were assessed for relevant studies.

## Results

### Search outcome

**Fig. 1:**
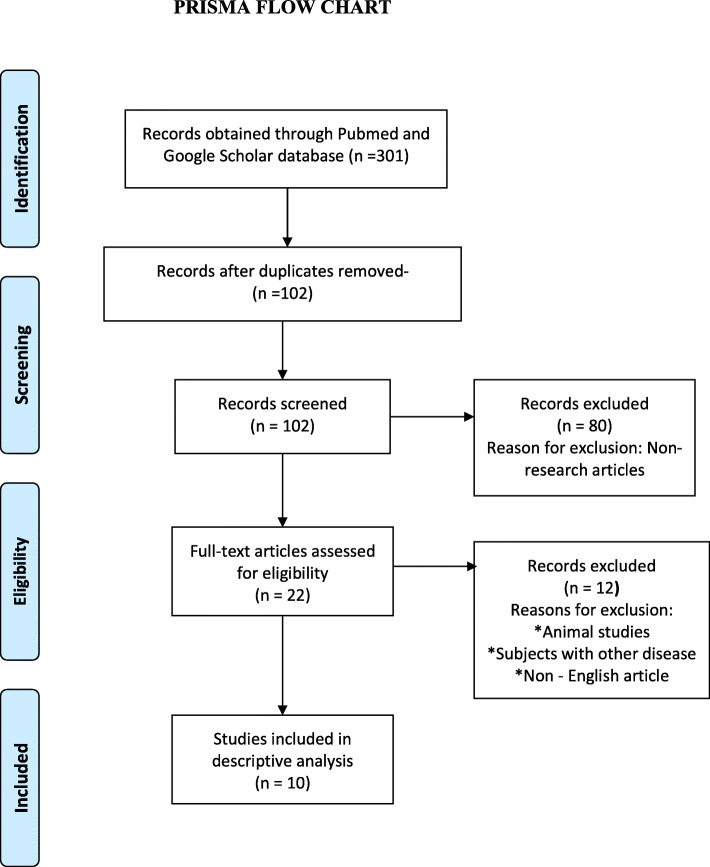
PRISMA flow chart

Three hundred one articles were obtained after using the mentioned literature search strategy. This included all types of articles. These articles were manually analyzed to rule out studies on the association of CHIP mutations with diseases other than ASCVD. After a thorough screening of abstracts and titles, 121 articles were manually selected. After excluding the duplicates, a total of 102 articles were found. Non-research articles and articles of not meeting our search criteria were excluded resulting in 22 studies to be included for full-text screening. Out of 22 studies, 11 studies were done exclusively on animals, 1 study was a pre-print and non-peer-reviewed article. Hence, these 12 studies were excluded for further analysis. The remaining 10 studies were used for the final descriptive analysis of our systematic review (Fig. 1).

Almost all the studies were published between 2017 and 2021. Out of 10 articles selected, 7 studies were cohort and 3 were case-control studies. All the articles analyzed the prevalence of CHIP mutation and its association with CVD. The methodology adopted for the identification of CHIP mutations in the selected studies included—whole-exome sequencing (*n* = 3), whole-genome analysis (*n* = 1), transcriptome profiling analysis (*n* = 1), whole-genome analysis (*n* = 1), and single-cell RNA-sequencing (*n* = 1).

### Description about the studies

Bick et al. [18] used high-coverage whole-genome sequences from 97,691 participants from the NHLBI TOPMed program and identified 4229 individuals with CHIP. They further identified associations of blood cell, lipid, and inflammatory traits specific to different CHIP gene mutations. Abplanalp et al. [19] studied 8 patients with severe degenerative aortic valve stenosis, 6 with chronic post-infarction heart failure, and 3 healthy controls. They used a single-cell RNA sequencing technique. The mean CHIP-driver gene variant allele frequency reported was 4.2% for DNMT3A and 14.3% for TET2.

Exome sequencing analysis was performed in two studies using the samples from UK BioBank.

One study was performed on 35,416 individuals without prevalent CVD and reported that 3% of subjects carried CHIP mutations. After 6.9 years of the follow-up period, they identified increased CVD risk (hazard ratio (HR) − 1.27%) in subjects with CHIP mutations (Bick et al. [20]). The other study was performed on postmenopausal women by Honigberg et al. [21]; 11,495 samples (40–70 years) were obtained from UK BioBank and 8111 samples (50–79 years) were obtained from Women’s Health Initiative (WHI). The study reported that among postmenopausal middle-aged women, CHIP was independently associated with incident coronary artery disease (HR associated with all CHIP: 1.36). Jaiswal et al. [22] performed a whole-exome analysis in 4726 patients with CHD and 3529 controls. They found that individuals with CHIP mutations are at 1.9 times more risk of developing CHD compared with non-carriers. They further evaluated TET2 knockout mice which showed increased expression of many chemokine and cytokine genes.

Out of the 4 studies that used deep sequencing analysis, one of the studies analyzed bone marrow-derived mononuclear cells from 200 patients with chronic heart failure and found 47 mutations in 38 of 200 patients with CHF (18.5%). The somatic mutations most commonly occurred in the DNMT3A, TET2, KDM6A, and BCOR. After a median follow-up of 4.4 years, 53 patients died, and 23 patients required hospitalization for heart failure. Poor long-term clinical outcomes for patients with either DNMT3A or TET2 mutations compared with non-CHIP carriers (Dorsheimer et al. [23]) were noted. The second study (Figal et al. [24]) analyzed 62 patients with heart failure (HF) and left ventricular ejection fraction (LVEF) < 45%. They reported that patients with mutations in either DNMT3A or TET2 exhibited an accelerated HF progression in terms of death (hazard ratio: 2.79).

Cremer et al. [25] analyzed 419 patients with chronic heart failure and reported a total of 223 mutations in 154 of 419 patients in their study. Mutations were most commonly found in DNMT3A and TET2. Higher mortality rates were observed in patients with CHIP mutations.

A study from Montreal Heart Institute Biobank by Busque et al. [26] analyzed 1359 CAD patients and 528 controls. It reported the presence of CHIP mutations in 427 of the 1887 subjects. The most frequently mutated genes were DNMT3A (11.6%) and TET2 (6.1%). Individuals with CHIP mutations showed higher hs-CRP levels compared with their noncarriers.

Transcriptome profiling analysis of peripheral blood mononuclear cells 6 patients with heart failure (HF) harboring DNMT3A CH-driver mutations and 4 patients with HF and no DNMT3A mutations by single-cell RNA-sequencing. Monocytes of HF patients carrying DNMT3A mutations demonstrated a significantly increased expression of inflammatory genes compared to monocytes derived from HF patients without DNMT3A mutations. The study concludes that the presence of DNMT3A mutations exhibit a highly inflamed transcriptome, which may contribute to the aggravation of chronic heart failure (Abplanalp et al. [27]). Descriptive details about the studies are presented in Table 2.

**Table 2:** Descriptive data of original articles reviewed

Year	Authors	Sample size	Study design	Outcome
2021	Wesley Tyler Abplanalp et al. [27]	10	Cohort	• Presence of DNMT3A mutation in the monocytes of heart failure patients has increased the expression of inflammatory genes which might aid the provoke the occurrence of chronic heart failure.
2021	Michael C. Honigberg et al. [21]	19,606	Cohort	• Association between natural premature menopause and CHIP = *P* = 0.001 • DNMT3A mutation was observed to be significantly associated with premature menopause • Natural premature menopause might be considered as a risk signal for developing CHIP or CHIP-associated CVD
2021	Domingo A. Pascual-Figal et al. [24]	62	Cohort	• Presence of DNMT3A or TET2 mutations might accelerate HF progression in terms of death (*P* = 0.008)
2020	Alexander G. Bick et al. [18]	97,691	Cohort	• > 75% of CHIP mutations in DNMT3A, TET2, and ASXL1. • 15% of CHIP mutations were in PPM1D, JAK2, SF3B1, SRSF2, and TP53
2020	Alexander G. Bick et al. [20]	35,416	Cohort	• CHIP associated with increased CVD event risk (*P* = 0.019) • Presence of large CHIP mutations adjusted for hSCRP value showed increase in CVD event risk (*P* = 0.0016) • Presence of rs1880241 shows reduced CVD events in large CHIP carriers (*P* = 0.025) • IL6R p.Asp358Ala allele reduces CVD event risk in individuals (*p* = 0.047)
2020	Wesley Tyler Abplanalp et al. [19]	17	Case-control	• Patients with DNMT3A or TET2 CHIP mutations can be categorized for high risk for adverse outcomes of COVID-19 • SARS-CoV-2 patients could be tested for the presence of DNMT3A or TET2 CHIP-driver sequence variations to provide personalized treatment strategies such as IL-6 or IL-6R antagonists to mitigate CRS
2020	Lambert Busque et al. [26]	1887	Case-control	• hs-CRP was significantly higher in CHIP carriers (*P* = 0.009) • DNMT3A CHIP mutation carriers had higher hs-CRP (*P* = 0.04)
2020	Sebastian Cremer et al.^25^	419	Cohort	Patients with two or more CHIP mutations have increased mortality risk (*P* < 0.001) as compared to patients with a single CHIP mutation or individuals without mutations. (*P* < 0.001).
2019	Lena Dorsheimer et al. [23]	200 CHF	Cohort	• DNMT3A ( 7% of patients); TET2 (4.5% of patients), KDM6A (2 % of patients), BCOR (1.5 % of patients) • Significantly worse long-term clinical outcome observed in patients with DNMT3A/TET2mutations
2017	S. Jaiswal et al. [22]	8255	Case-control study	The presence of CHIP mutation nearly doubled the risk of coronary heart disease in humans and mice with accelerated atherosclerosis.

CHIP mutations found in DN*MT3A*, *TET2*, *ASXL1*, *TP53*, *JAK2*, and *SF3B* genes are associated with an increased risk of having CVD. Most of the genes implicated in CHIP are involved in methylation and demethylation proteins governing DNA structural properties.

To further study the functionality and the interactions of the mutated genes, online databases were used to establish the protein-protein interaction. Functionality assessments of the genes were analyzed using profiler software. g: Profiler (http://biit.cs.ut.ee/gprofiler/) is a public web server for characterizing and manipulating gene lists resulting from mining high-throughput genomic data. g: GOSt functional enrichment analysis maps genes to known functional information sources and detects statistically significantly enriched terms. Functionality assessment analysis of the genes revealed significantly enriched BioGRID interactions for chromatin organization and modification, histone modification and kinase activity, euchromatin, and epigenetic regulation of gene expression. Human phenotype ontology revealed significant interaction with events related to CVD (Table 3).

**Table 3:** Significantly enriched BioGRID interactions of most commonly mutated genes indicating the molecular function, biological process, cellular component, and biological pathways

Description	Term ID	Corrected ***P*** value
**Biological process**		
Covalent chromatin modification	GO:0016569	8.13797E−05
Chromatin organization	GO:0006325	0.001237802
Bone marrow development	GO:0048539	0.002607555
Histone modification	GO:0016570	0.006671044
Chromosome organization	GO:0051276	0.009673322
**Molecular function**		
Histone kinase activity (H3-Y41 specific)	GO:0035401	0.049878314
Histone tyrosine kinase activity	GO:0035400	0.049878314
**Cellular component**		
Euchromatin	GO:0000791	0.009699567
U11/U12 snRNP	GO:0034693	0.049903619
**Biological pathways (reactome)**		
Epigenetic regulation of gene expression	REAC:R-HSA-212165	0.010116540
Amine ligand-binding receptors	REAC:R-HSA-375280	0.008152302
**Human protein atlas**		
Lymph node; non-germinal center cells [high]	HPA:0310443	0.033971887
**Human phenotype ontology**		
Abnormal cellular immune system morphology	HP:0010987	0.002011691
Abnormal leukocyte morphology	HP:0001881	0.002011691
Chest pain	HP:0100749	0.00232273
Abnormal platelet morphology	HP:0011875	0.002471705
Abnormal immune system morphology	HP:0032251	0.00256098
Abdominal pain	HP:0002027	0.002830113
Arterial thrombosis	HP:0004420	0.003992637
Weight loss	HP:0001824	0.00406694
Abnormal number of granulocyte precursors	HP:0012137	0.004170906
Megaloblastic erythroid hyperplasia	HP:0200143	0.004170906

**Fig. 2:**
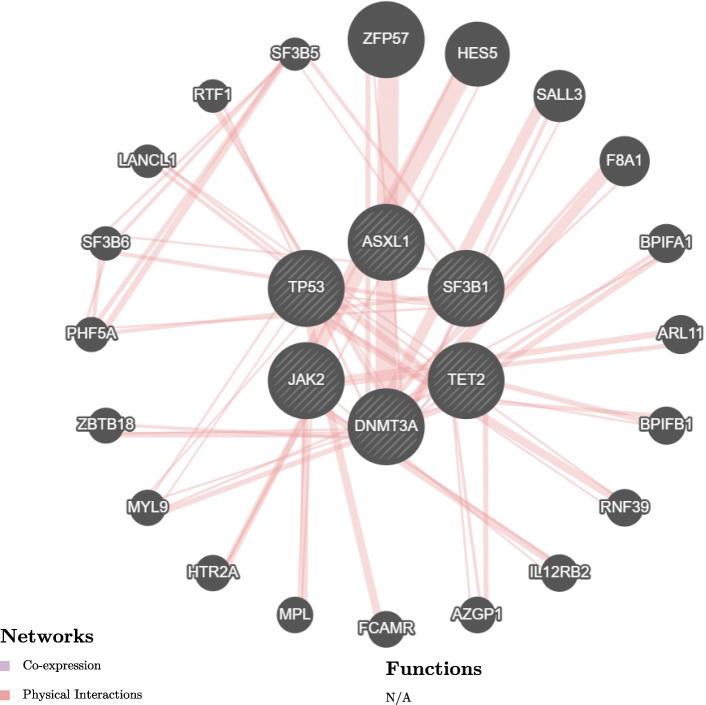
Network analysis of the *DNMT3A*, *TET2*, *ASXL1*, *TP53*, *JAK2*, and *SF3B1* genes using GeneMania

To predict the possible gene association network and gene functions of the most commonly mutated genes in silico, analyses were performed using the GeneMANIA (Gene Function Prediction using a Multiple Association Network Integration Algorithm—an integrated interaction network program that predicts gene functions and possible interaction networks using many large publicly available datasets including protein-protein and genetic interaction networks (Fig. 2)). Analysis revealed that the association network members were linked through these networks: co-expression 53.08% and physical interactions 46.92%.

## Discussion

The main findings of our systematic review are as follows:

1. There is an association between CHIP and CVD.
2. The most commonly described CHIP mutations in patients with CVD are *DNMT3A*, *TET2*, *ASXL1*, *TP53*, *JAK2*, and *SF3B.*
3. We further analyzed the most commonly mutated CHIP genes using bioinformatics tools. Protein function and interaction analysis were performed using the g: Profiler and GeneMANIA online tools. The results revealed significant biogrid interactions for molecular functions, biological processes, and biological pathways. Interaction analysis showed significant physical and co-expression interactions.

It has been well established that the presence of CHIP mutations has an increased risk of malignancy diagnosis. Earlier studies on murine models on functional analysis of these mutations indicated that heterozygous or homozygous loss of function of TET allele, have larger atherosclerotic plaques than the controls [28, 29]. Loss of function of *CHIP* mutation*s* has been found to affect self-renewal and differentiation of HSCs into myeloid lineages. In addition, clonal expansion of such mutant cells may interfere with the production of macrophages, endothelial cells involved in the vascular repair process.

Despite having more than 30% of the world population in India, no data regarding the same is available from India. Indian patients develop CAD is at least a decade earlier than their western counterparts. It is too early to say that CHIP mutations are the sole contributor to the same. However, we believe their association could not be meager as shown by Jaiswal et al. in their study which studied patients from Pakistan [30]. We are still in the phase of understanding CHIP in patients with cardiovascular diseases; there is a lack of evidence that helps us in managing patients with CHIP. Understanding potential mechanisms will help us to provide specific therapeutic targets. Though no robust evidence is available in the management of such patients with CHIP and cardiovascular diseases, poorer outcomes seen in such patients with CAD, patients with heart failure, and patients with valvular heart diseases enunciate that an aggressive lifestyle modification including smoking cessation with control of risk factors like blood pressure, blood sugar, and cholesterol should be strongly considered as the first-line of therapy till we identify specific therapies that target CHIP mutations directly.

The survival of individuals has increased due to the advancement of healthcare leading to more and more elderly patient populations in our society. As aging is the most important factor to be associated with CHIP, we will see more patients with CHIP mutation in the future. Whole-genome sequencing is also made commercially available. Public with interest in knowing their genetic risk may get their genetic sequencing and end up knowing their CHIP status incidentally. Hence, the medical community has understood the importance of identifying CHIP mutation and has started offering personalized CHIP testing in patients with premature CAD or who develop CAD without any traditional risk factors in selected centers of western countries [29]. A multi-disciplinary approach with greater cooperation between oncologists, cardiologists, and geneticists is warranted to avoid undue apprehension that can happen due to incidental diagnosis of CHIP status. Proper genetic counseling is strongly encouraged before going for deep sequencing to identify CHIP mutations to help patients to circumvent excessive worries.

## Conclusion

We conclude that there exists a strong association between CHIP gene mutations (*DNMT3A*, *TET2*, *ASXL1*, *TP53*, *JAK2*, and *SF3B)* and CVD. At present, no data about such somatic mutations in patients with CVD is available from India. We intended to investigate the same in our patients with CAD in our pilot research study which is funded by the Indian Council of Medical Research (ICMR). The most important limitation in doing such a cutting-edge research study that can potentially open a new pandora box in the management of patients with CAD is the cost involved in doing deep sequencing. A larger prospective study with multi-center involvement from India will need huge funding from public agencies.

## Supplementary Information

**Additional file 1.** Gene Descriptions. The supplementary article provides description about the genes identified through network analysis using GeneMania.

## Data Availability

Supplementary data available.

## Abbreviations

BMI: Body mass Index
CHIP: Clonal hematopoiesis of indeterminate potential
CVD: Cardiovascular diseases
DNA: Deoxyribonucleic acid
*JAK2*: Janus kinase 2
*TET2*: Tet Methylcytosine Dioxygenase 2
*DNMT3A*: DNA Methyltransferase 3 Alpha
*ASXL1*: Additional Sex Combs Like Transcriptional Regulator 1
*SF3B*: Splicing factor 3B subunit 1
