# Supplementary figures and images for: Clonal hematopoiesis of indeterminate potential (CHIP) and cardiovascular diseases—an updated systematic review
Source: J Genet Eng Biotechnol. 2021 Jul 19;19:105. doi: 10.1186/s43141-021-00205-3 (PMC8287286; doi:10.1186/s43141-021-00205-3)

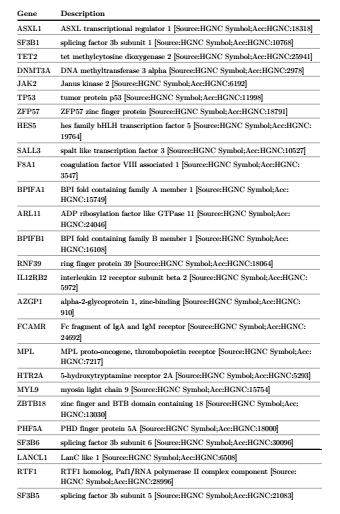

Supplement: Supplementary file 1 — Additional file 1. Gene Descriptions. The supplementary article provides description about the genes identified through network analysis using GeneMania. [file 43141_2021_205_MOESM1_ESM.jpg]
